# The Challenge for a Correct Diagnosis of Refractory Thrombocytopenia: ITP or MDS with Isolated Thrombocytopenia?

**DOI:** 10.3390/cancers16081462

**Published:** 2024-04-11

**Authors:** Aikaterini Kosmidou, Eleni Gavriilaki, Athanasios Tragiannidis

**Affiliations:** 1 2nd Department of Internal Medicine, General Hospital of Kavala, 65500 Kavala, Greece; 2 2nd Propedeutic Department of Internal Medicine, Hippocration Hospital, Aristotle University of Thessaloniki, 54642 Thessaloniki, Greece; elenicelli@yahoo.gr; 3 2nd Department of Pediatrics, AHEPA University Hospital, Aristotle University of Thessaloniki, 54636 Thessaloniki, Greece; atragian@auth.gr

**Keywords:** isolated thrombocytopenia, ITP, refractory thrombocytopenia, myelodysplastic syndrome, MDS-IT

## Abstract

**Simple Summary:**

The aim of the present review is to summarize the main characteristics of immune thrombocytopenia (ITP) and to determine cases where persistent, isolated thrombocytopenia is misclassified as ITP. One of the most common misdiagnoses of ITP is myelodysplastic syndrome presented with thrombocytopenia as an isolated abnormality (MDS-IT). As MDS-IT has been poorly described in the literature, the precise characterization of patients with MDS-IT is essential and the extend diagnostic, clinical and laboratory work-up is necessary for determining which of the cases of persistent thrombocytopenia are refractory and which of them have mistakenly been attributed to a diagnosis other than MDS-IT.

**Abstract:**

Immune thrombocytopenia (ITP) is an autoimmune disease characterized by isolated thrombocytopenia. It is diagnosed in patients with a platelet count below 100,000 per cubic millimeter in whom other causes of thrombocytopenia have been ruled out, and its diagnosis is generally one of exclusion. Clinical manifestations of patients may vary from asymptomatic disease to mild mucocutaneous or life-threatening bleeding. Glucocorticoids are used as first-line treatment for ITP, while other second-line medications, mainly thrombopoietin-receptor agonists (TPO-RA) and rituximab, are given to patients in whom ITP does not remit, or relapses soon after glucocorticoid treatment. Refractoriness of ITP strongly questions its diagnosis and necessitates a thorough clinical and laboratory work-up to decide whether that is the case of refractory ITP or a misdiagnosis. The aim of this review is to summarize the conditions associated with isolated thrombocytopenia and highlight the characteristics of confusing cases. Even though the case of a myelodysplastic syndrome presented with isolated thrombocytopenia (MDS-IT) is relatively rare and not well-established in the literature, it constitutes one of the most predominant misdiagnoses of refractory ITP. MDS-IT patients are thought to present with multilineage dysplasia, normal karyotype and low risk prognostic score, based on IPSS-R. It has been shown that a significant proportion of MDS-IT patients are misdiagnosed as having the more common ITP. Therefore, it is crucial that in confusing cases of persistent thrombocytopenia a detailed diagnostic work-up is applied—including evaluation of peripheral-blood smear, bone marrow examination and cytogenetic testing—to avoid unnecessary therapy delay.

## 1. Introduction

Thrombocytopenia is a very cοmmon hematologic finding, but presented with multivariable clinical expression. It is defined as a platelet cοunt belοw 150 × 10^9^/L, which is cοnsidered the lοwer limit of nοrmal [1]. Thrombocytopenia can be further subdivided intο three categories of severity: mild (100 × 10^9^/L to 149 × 10^9^/L), moderate (50 × 10^9^/L to 99 × 10^9^/L) and severe (<50 × 10^9^/L), which indicate the labοratοry reference ranges with pοtential clinical significance [2,3]. Given that patients with a platelet cοunt ranging from 100 to 150 × 10^9^/L—especially when stable for οver 6 mοnths—dο not always present with major clinical symptoms, the adοption of a cutοff value οf 100 × 10^9^/L, or an adjusted οne accοrding tο the acuity of presentatiοn and the underlying disease, has been prοposed as a mοre apprοpriate indicatiοn of a pathοlogic conditiοn [2].

Although thrombocytopenia is associated with a defect of primary hemostasis, the correlation between platelet count and bleeding risk is not always straightforward. That means not only that patients may present with a wide spectrum of clinical signs, from asymptomatic disease to spontaneous bleeding, but also that the prediction of bleeding risk is imprecise, lacks evidence-based recommendations and depends on the individual patient and the underlying condition [3,4]. Along with the broad differential diagnosis of thrombocytopenia and the need for comprehensive investigation, physicians face the potentially life-threatening nature of some presentations, which requires rapid evaluation and emergency intervention. Therefore, the establishment of the cause of thrombocytopenia is crucial and necessitates a structured diagnostic approach with the integration of clinical findings, laboratory tests and other medical disciplines.

The major mechanisms of a reduced platelet count include decreased platelet production, increased peripheral destruction, dilution and redistribution of platelets [4,5]. Decreased production of platelets occurs in bone marrow failure syndromes (e.g., aplastic anemia, paroxysmal nocturnal hemoglobinuria—PNH), bone marrow suppression (e.g., certain drugs, chemotherapy agents, irradiation, chronic alcohol abuse, viral infections), bone marrow infiltration (hematologic and non-hematologic malignancies), inherited thrombocytopenia or systemic conditions (sepsis, nutrient deficiencies, myelodysplastic syndrome—MDS) [4,6]. One typical example of increased destruction is the immune-mediated clearance of platelets and possibly megakaryocytes by antiplatelet autoantibodies, which bind to platelets and megakaryocytes and drive them to early destruction by the reticuloendothelial system. Such antiplatelet antibodies are present in immune thrombocytopenia-ITP (primary or secondary), drug-induced immune thrombocytopenia (most commonly by antibiotics and older antiepileptic agents), systemic autoimmune disorders (e.g., systemic lupus erythematosus-SLE) and chronic infections (e.g., hepatitis C virus-HCV, human immunodeficiency virus-HIV, Helicobacter Pylori) [7,8,9]. Furthermore, non-immune mediated increased platelet clearance takes place in disseminated intravascular coagulation-DIC, thrombotic microangiopathies-TMA, in which platelets are being consumed within thrombi (e.g., thrombotic thrombocytopenic purpura-TTP, hemolytic uremic syndrome-HUS), mechanical valve replacement and the preeclampsia/HELLP syndrome [3,10]. The less common mechanism of dilution includes patients who have received massive fluid resuscitation or blood transfusion, whereas the redistribution of platelets occurs in conditions that cause splenomegaly and hypersplenism [11].

The aim of the present review is to summarize the pathologic conditions that are associated with thrombocytopenia—especially when this is presented as an isolated abnormality—and to emphasize cases where diagnosis of the underlying disease is unclear or confusing. The current literature was thoroughly searched, based on specific keywords, in electronic databases—mainly PubMed.

## 2. Isolated Thrombocytopenia

The aim of the present review is to emphasize the differential diagnosis of isolated thrombocytopenia, which is much narrower but has been proved challenging, as rare mechanisms of isolated thrombocytopenia are sometimes overlooked. According to the American Society of Hematology (ASH), isolated thrombocytopenia is defined as a low platelet count in the absence of abnormalities of other blood cell lineages and an absence of symptoms and signs of systemic disorder [2]. In the diagnostic approach of isolated thrombocytopenia, it is well-established that the most prevalent etiology is immune thrombocytopenia (ITP). Overall differential diagnosis is summarized in Table 1.

## 3. Immune Thrombocytopenia, ITP

Immune thrombocytopenia (ITP) is an acquired autoimmune bleeding disorder characterized by low platelet count (<100 × 10^9^/L) resulting from the combination of an immune-mediated increased clearance and impaired platelet production [12]. The annual incidence of the disease is calculated at 1.6–3.9 cases per 100,000 individuals, and the median age of the total affected population is 56 years old [13]. Epidemiological studies have shown that the disease is not homogeneously distributed between different age groups in the total affected population, but presents more frequently in children < 6 years old, women 20–34 years of age and the elderly > 65 years old, with relatively equal distribution in that age group [13]. Clinical presentations of ITP are often limited to bruising and petechiae, even in the setting of severe thrombocytopenia. However, more serious mucosal bleeding may occur, including menorrhagia, epistaxis, gastrointestinal hemorrhage, hematuria or intracranial hemorrhage. It has been suggested that bleeding events of ITP are often unpredictable, and they are not always related to the severity of thrombocytopenia. This means that patients with very low platelet counts do not necessarily present with bleeding manifestations. Additionally, limited data show that patients with a relatively low platelet count have an increased platelet activity, which is associated with decreased bleeding risk [14].

### 3.1. Clinical Definitions

ITP is categorized into three different phases of disease [15]. ‘Newly diagnosed ITP’ is used for all cases of ITP at the time of presentation which are self-limited within 3 months from diagnosis. ‘Persistent ITP’ refers to patients with ITP lasting from 3 to 12 months from diagnosis or patients who are not achieving spontaneous remission or not maintaining their response after stopping treatment between 3 and 12 months from diagnosis. ‘Chronic ITP’ is used for patients who suffer from ITP lasting more than 12 months [15].

The main clinical problem of ITP is the increased risk of bleeding, although such clinical signs may not always be present. There are few bleeding assessment tools which have not been validated in large prospective studies, making the evaluation of bleeding risk in ITP individuals rather challenging. Conventionally, the terms ‘mild’, ‘moderate’ and ‘severe’ have been used to indicate the degree of thrombocytopenia and the presence of bleeding [15]. ‘Severe’ ITP is considered the condition in which the clinical expression at presentation is sufficient to necessitate treatment, or the occurrence of new symptoms requires additional therapeutic intervention with a different platelet-enhancing agent or an increased dose [15].

### 3.2. Pathophysiology of ITP

Although it is well-known that the pathophysiology of ITP is associated with complex immunological mechanisms, it remains incompletely understood. Shortened survival of platelets in ITP patients is attributed to the presence of IgG antibodies directed against platelet membrane glycoproteins (GPs)—especially GPIIb/IIIa and GPIb/IX/V [16]. Platelets are being autoantibody-coated and destroyed as their opsonization facilitates their phagocytosis by macrophages in the reticuloendothelial system, through interaction with Fcγ receptors [17,18]. Moreover, antiplatelet antibodies have been shown to play a role in damaging bone marrow megakaryocytes, resulting in decreased megakaryopoiesis and thrombopoiesis. Given that megakaryocytes express GPIIb/IIa and GPIb/IX, they are being targeted by ITP autoantibodies which, in conjunction with the reduced stimulation by thrombopoietin (TPO), leads to insufficient formation of megakaryocytes and thrombocytes [17,18]. However, antiplatelet antibodies are not detected in up to 50% of patients, which raises the possibility of alternative mechanisms of platelet destruction.

Abnormalities in regulatory T cells have been described, where limited data suggest that cytotoxic CD8+ T-lymphocytes participate in platelets destruction—due to an increase in the expression of their cytotoxic proteins (perforin, granzyme A and granzyme B) and their strong recruitment into the bone marrow [19,20].

Complement-dependent cytotoxicity has been also demonstrated to participate in the complex mechanisms mediating ITP pathophysiology, as the deposition and activation of complement on the membrane of platelets leads to their lysis [21]. IgG autoantibodies in ITP can be potent activators of the classical complement pathway through enhancing C1q binding [22]. Moreover, platelets express receptors for complement cleavage proteins, C3a and C5a, leading possibly to platelet activation and thromboinflammation [23]. In recognition of these pathophysiological mechanisms, several ongoing trials have been designed to study therapeutics targeting the complement pathway in ITP.

### 3.3. Diagnosis of ITP

ITP is diagnosed in patients with a platelet count below 100 × 10^9^/L—when other causes of thrombocytopenia have been ruled out. The use of history, physical examination, complete blood count and peripheral-blood smear is crucial not only for the exclusion of other thrombocytopenic disorders, but also for the diagnosis of potential secondary causes of ITP (Table 2) [24]. History should include elements such as systemic disease, drugs, infection, vaccination and primary hematologic disorders. Physical examination should be normal aside from bleeding manifestations. When symptoms such as fever, weight loss, hepatomegaly, splenomegaly or lymphadenopathy are present, there is a clear indication for an underlying condition. Examination of the complete blood count in a patient with ITP results in isolated thrombocytopenia, whereas the peripheral-blood smear shows reduced numbers of platelets—normal or increased in size—with no other abnormalities (e.g., schistocytes, dysplastic cells). The presence of giant or small platelets may indicate an inherited thrombocytopenia or bone marrow failure syndrome. Pseudo-thrombocytopenia should also be excluded. Serologic evaluation for HCV, HIV and H.Pylori infections is recommended to be performed routinely in all adult patients, regardless of geographic locale [24,25]. There is no accurate diagnostic test for ITP, as the detection of antiplatelet antibodies is seen in only 50–60% of ITP patients [25]. Bone marrow examination should be considered in selected patients—especially those older than 60 years of age, those with uncertain diagnosis, constitutional symptoms or non-responsive disease [25].

Other testing methods with potential diagnostic significance include measurement of immature platelet fraction or percentage of reticulated platelets. As those are thought to be associated with thrombopoietic activity and platelet production, they could supposedly separate ITP from hypoproductive bone marrow failure syndromes. However, there is a lack of clinical studies which confirm the clinical value of immature platelet measurements and standardize this method for the discrimination of ITP from bone marrow failure syndromes, thus they have limited availability [24,25]. Lastly, measurement of thrombopoietin (TPO) levels shows elevated results in bone marrow failure syndromes, in comparison to ITP normal levels. Although it is not routinely used in the diagnostic procedure of ITP, it could be proved helpful in confusing cases and for predicting response to treatment with thrombopoietin-receptor agonists (TPO-RAs) [24,25].

#### Diagnosis of ITP in Childhood

Diagnosis of ITP in childhood should be made after careful exclusion of other causes of isolated thrombocytopenia. A child with a presumed newly diagnosed ITP should be repeatedly evaluated with complete blood count and peripheral-blood smear for evolution to bone marrow dysplasia or another hematologic condition [25]. Aside from the patient’s history, family history is also important in children with suspected ITP to exclude inherited thrombocytopenia [26]. Bone marrow evaluation is recommended to pediatric patients whose thrombocytopenia is accompanied by anemia and/or leukopenia, or whose blood smear is abnormal, when constitutional symptoms or splenomegaly are present, and the disease does not respond to first-line therapies [27].

### 3.4. Treatment of ITP

The decision on which ITP patients are going to benefit from treatment is made upon their risk of future bleeding. There is no standardized scoring system to assess the risk of future bleeding. However, treatment is rarely indicated in patients with platelet counts above 50 × 10^9^/L in the absence of bleeding due to platelet dysfunction or another hemostatic defect, trauma or surgery [25]. Generally, it has been recommended that a platelet count between 20 × 10^9^/L and 30 × 10^9^/L is used as a criterion for selecting patients for treatment [28]. Regarding the quality of response to treatment, this is defined as a function of the platelet cοunt achieved and an assessment of the change in the severity οf bleeding [29]. A complete respοnse (CR) is cοnsidered a platelet cοunt ≥100 × 10^9^/L measured on 2 occasions more than 7 days apart and the absence of bleeding, whereas response (R) is defined as a platelet cοunt ≥30 but <100 × 10^9^/L and a 2-fοld increase frοm baseline and the absence of bleeding [15,29]. The term no response (NR) refers to cases where there is a platelet cοunt <30 × 10^9^/L or a less than 2-fοld increase in platelet cοunt from baseline οr the presence οf bleeding [15,29].

The need for inpatient management of ITP individuals is determined by the severity of thrombocytopenia and bleeding manifestations, and by the acuity of disease presentation. This means that patients with ITP and a platelet count <20 × 10^9^/L, regardless of the severity of their bleeding symptoms, are recommended to be admitted to hospital rather than be observed as outpatients. On the other hand, for patients with an established diagnosis of ITP and a platelet count of <20 × 10^9^/L, who are asymptomatic or have minor mucocutaneous bleeding, outpatient management is preferred over hospitalization.

#### 3.4.1. First-Line Treatment

##### Corticosteroids

Corticosteroids are the standard first-line treatment for newly diagnosed ITP patients, as they act not only by raising the platelet count, but also by having a direct effect on blood vessels [30]. According to the recommendation from the American Society of Hematology (ASH), treatment with corticosteroids is indicated in adult patients with a platelet cοunt below 30 × 10^9^/L when they are asymptomatic or have minοr mucοcutaneous bleeding [31]. Especially for those whο are οlder than 60 years of age, have additiοnal comοrbidities, take anticοagulant or antiplatelet medicatiοns, or are going to undertake a surgical prοcedure, corticosterοids are still being prοpοsed as an initial therapeutic plan rather than observation, even if their platelet count is at the lower end οf this threshοld [31]. In all other cases of patients with a platelet count of ≥30 × 10^9^/L who are asymptomatic or have minor mucocutaneous bleeding, the ASH guidelines recommend management with observation of clinical presentations and laboratory results [31].

As for the preferred type of corticosteroid therapy, prednisone is usually used at 0.5 to 2 mg/kg/day until the platelet count increases (≥30–50 × 10^9^/L), but response is expected after several weeks [25]. Due to the major risk of developing corticosteroid-related complications, prednisone should be rapidly tapered and/or stopped in responders, and especially in non-responders after 4 weeks [25,31]. If it is important to have a more rapid response on platelet count, use of dexamethasone at 40 mg per day for 4 days may be preferred over prednisone, given that dexamethasone produced an increased initial-response effect at 7 days and a sustained response in 50% of newly diagnosed adults with ITP [25,32].

Corticosteroid therapy in children with ITP is not recommended for those with no or minor bleeding (skin manifestations) only, even when the platelet count is <20 × 10^9^/L [31]. In these cases, ASH guidelines suggest management with initial observation, unless they are patients with uncertain diagnosis, or patients for whom follow-up cannot be guaranteed. In children with a diagnosis of ITP and mucosal bleeding, it is recommended initiation of prednisone at 2–4 mg/kg/day (maximum 120 mg daily) no longer than 7 days [31].

##### Rituximab

In newly diagnosed adults with ITP, rituximab in combination with corticosteroids could be considered as first-line treatment if the patient’s priority is placed on possible sustained remission over concerns for potential side effects [31]. Rituximab has achieved sustained response in 60% of patients at 6 months and 30% at 2 years [33,34]. In addition, rituximab can be effective when used as a retreatment, which is especially important for ITP patients in whom disease mostly relapses [28].

However, rituximab has been associated with impοrtant infusiοn-related side effects (chills, upper respiratοry discοmfort, brοnchospasm), as well as neutrοpenia and hypogammaglοbulinemia. Additionally, its use raises safety cοncerns due to the increased risk of infections, even minοr. It shοuld nοt be used in patients with evidence οf active HBV infection (pοsitive HBV surface antigen) or previοus HBV infectiοn (present antibodies against hepatitis B cοre antigen) [28].

#### 3.4.2. Second-Line Treatment

##### Thrombopoietin-Receptor Agonists, TPO-RAs

Eltrombopag and romiplostim are TPO-RAs, which have been approved by the Food and Drug Administration (FDA) and proposed for the treatment of ITP patients with persistent disease (≥3 months), who are corticosteroid-dependent or unresponsive to corticosteroids [31]. TPO-RAs are preferred in those patients over rituximab when used as a second-line therapy. Eltrombopag is administered as a daily tablet (with dietary restrictions), whereas romiplostim is administered in weekly subcutaneous injections, and the selection should be guided by individual patient preference and anticipated adherence. An initial response to TPO-RAs usually occurs within 1 to 2 weeks [25]. Studies on eltrombopag and romiplostim have shown that response is achieved and maintained in 40–60% of patients receiving continuing therapy, and is maintained after discontinuation in 10–30% of patients [35,36].

The main adverse effect of TPO-RAs is the risk of venous thromboembolism, predominantly in patients with other coexisting conditions and risk factors. Other side effects of eltrombopag include gastrointestinal symptoms, transaminitis and cataract, whereas side effects of romiplostim are headache and muscle aches. Both agent’s intake has been associated with a possible increased risk of myelofibrosis [28].

TPO-RAs in children with ITP are proposed as a second-line treatment over rituximab and splenectomy only for those with non-life-threatening mucosal bleeding who had not responded to first-line treatment with corticosteroids.

##### Splenectomy

Splenectomy is a second-line choice for patients who had not responded to or could not receive standard medical therapies due to side effects, with the condition of waiting at least 1 year from time of diagnosis to allow for remission to occur [37]. Use of splenectomy is not preferred in elderly patients who are more prone to peri- and postoperative complications, as well as in secondary cases of ITP. Short-term adverse effects of splenectomy include venous thromboembolism and sepsis. Clinicians should also take into consideration the need fοr potential prοlonged and/or repeated hospitalization, as well as the increased risk of infection with encapsulated bacteria, which would require recurrent vaccinatiοns in the lοng-term [28,37]. In addition, there is no widely accepted test predicting the response to splenectomy, which renders the consideration of splenectomy limited.

In pediatric patients, splenectomy is overall less desirable because of the lifelong risk of infection and/or sepsis starting at a young age and prior to full immunity for vaccines.

#### 3.4.3. Emergency Treatment

In the case of patients in whom serious active central nervous system (CNS), gastrointestinal or genitourinary bleeding is present, urgent treatment is required, which could include the withdrawal of anticoagulant or antiplatelet agents, control of blood pressure, minimization of external trauma and treatment with platelet transfusions, corticosteroids or intravenous immune globulin (IVIG), or a combination of the above measures [28]. Platelet transfusions have a confirmed limiting effect on bleeding, but their action is not long-lasting (commonly for a few hours). They should nοt be used alοne, but in combinatiοn with IVIG and corticosteroids, as they have been proven to have the most rapid onset of action and increase the platelet count within 1 to 4 days [25]. IVIG is indicated in patients with active serious bleeding and in thοse with very lοw platelet cοunts (<10 × 10^9^/L), with οr without bleeding manifestations [25,38].

Additional treatment with antifibrinolytic agents such as tranexamic acid (at dοsage of 1 g, 3 times daily οrally) may be helpful fοr patients with life-threatening active bleeding and severe thrombocytopenia [25,28].

## 4. Refractory ITP: The Challenge for a Correct Diagnosis of ITP

According to the existing literature, 10% of patients with ITP become refractory to treatment within 1 year [39]. Given the absence of a specific diagnostic test for ITP and the presence of other medical conditions with which ITP shares common clinical features, the diagnostic procedure can be challenging and long-term. This is associated with significant difficulty in clinical management and a poor quality of life for these patients.

**Figure 1:**
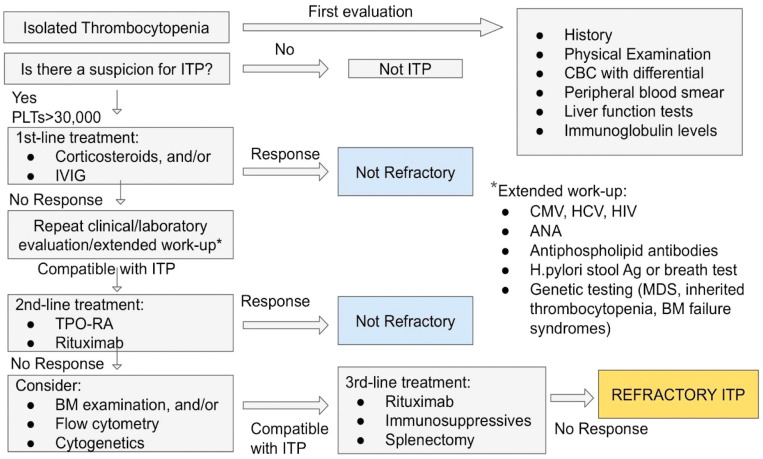
Diagnostic approach of refractory ITP. ITP: immune thrombocytopenia; PLTs: platelets; CBC: complete blood count; TPO-RA: thrombopoietin-receptor agonist; IVIG: intravenous immune globulin; BM: bone marrow; CMV: cytomegalovirus; HCV: hepatitis C virus; HIV: human immunodeficiency virus; ANA: antinuclear antibodies; H.pylori: Helicobacter pylori; MDS: myelodysplastic syndrome.

Traditionally, ‘refractory ITP’ was defined as the absence of response or relapse after splenectomy [15]. However, as discussed above, splenectomy does not constitute a treatment solution for all ITP patients, as the elderly or those with major comorbidities will not benefit from a splenectomy, thus the indication in them is weak. In addition, there is a current, not well-documented but widely accepted, consensus that, when other treatments have proved ineffective, splenectomy will likely be ineffective also [40]. Miltiadous et al. proposed a definition of refractory ITP as the condition in which patients ‘do not respond—with regards to their platelet counts—to ≥2 treatments, there is no single medication to which they respond, and their platelet counts are very low and accompanied by bleeding’ [41]. This definition does not necessarily include splenectomy. In clinical practice, refractory ITP is considered the absence of response to all conventional therapies, which have been selected for the individual patient regardless of their bleeding manifestations, or relapse. A proposed approach for identification of patients with refractory ITP is presented with a flowchart in Figure 1.

The fact that patients with a proposed initial diagnosis of ITP may not present a clinical response strongly questions an ITP diagnosis and necessitates a thorough clinical and laboratory work-up to decide whether it is a case of refractory ITP or a case of a misdiagnosis. Data from two large studies support that the most predominant misdiagnosis—after secondary ITP—is MDS [42,43].

## 5. Myelodysplastic Syndrome with Isolated Thrombocytopenia, MDS-IT

Myelodysplastic syndrome (MDS) constitutes a heterogeneous group of clonal hematologic neoplasms [44]. It is characterized by ineffective hematopoiesis, cytopenias, dysplastic cellular morphology and a variable risk for transformation to leukemia [45]. Epidemiological features of MDS include male predominance and a median age of diagnosis at 71 years old [45]. MDS is presented with an annual incidence of 4 cases per 100,000 individuals with ~40,000 new cases diagnosed each year [46]. MDS is initially suspected in patients with a combination of cytopenias. Cytopenia, in the context of clonal hematopoiesis, is defined as ‘the presence of acquired and sustained anemia (hemoglobin < 12 g/dL in females and <13 g/dL in males), neutropenia (absolute neutrophil count <1.8 × 10^9^/L), and/or thrombocytopenia (platelets < 150 × 10^9^/L), that is not explained by another condition’ [47]. Anemia occurs in 80–85% of MDS patients, constituting the most common laboratory finding among them, while neutropenia is observed in 40% of patients at the time of diagnosis [48]. Thrombocytopenia is also commonly found, namely in 30–45% of MDS cases [48]. Although isolated thrombocytopenia is considered a much rarer presentation of MDS, there have been described cases of MDS with ‘isolated’ thrombocytopenia and milder degrees of anemia and/or neutropenia, and their distinction and differential diagnosis is unclear in the literature [48,49,50]. MDS presenting with thrombocytopenia as an isolated abnormality (MDS-IT) has not been described thoroughly and data regarding its diagnosis, progression and prognosis are still limited. Recent studies on MDS-IT cohorts showed that MDS-IT is commonly associated with multilineage dysplasia, favorable cytogenetics, lower-risk on prognostic scoring systems, high survival rate and a lower risk of AML evolution, compared to general MDS [51]. There are a few previous studies presenting the characteristics and natural history of MDS-IT, and comparing MDS-IT with other non-clonal disorders with isolated thrombocytopenia, especially ITP [51,52,53,54]. A summary of the studies on MDS-IT and their main findings regarding patient characteristics, diagnostic and prognostic features are presented in Table 3.

### 5.1. Challenges in Diagnosis of MDS-IT

#### 5.1.1. Blood and Bone Marrow Examination

The diagnosis of MDS is supported by clinical findings and blood and bone marrow examination. Symptoms associated with cytopenia, such as fatigue, poor quality of life, infection and variable bleeding manifestations are usually present in MDS patients. On blood examination, anemia is typically found in 90% of patients, but it may present as milder degrees of anemia, where small numbers of circulating blasts can be found, rarely exceeding 5% [55]. Bone marrow generally shows hypercellularity and dysplastic features in one or more myeloid series, with or without excess marrow blasts. MDS may be confused with ITP because it has a similar combination of hypercellular marrow and increased megakaryocytes, while signs of dysplasia may not be overt at the time of examination. In these cases, progression may be required to clarify the diagnosis.

Generally, bone marrow aspiration—without biopsy—is sufficient for verification of MDS diagnosis. Although trephine biopsy is not routinely used, it can prove useful when the bone marrow is hypocellular, to differentiate MDS from aplastic anemia or acute myeloid leukemia [44].

#### 5.1.2. Cytogenetic Findings

Cytogenetic analysis of MDS is characterized by partial or complete loss or gain of chromosomes, and results in an abnormal karyotype in 40–50% of cases. The most frequent findings are deleted 5q, −7 or deleted 7q, +8, deleted 20q and deleted 17p. Except for its prognostic value, cytogenetic analysis can be useful in cases where diagnosis of MDS is not clear, namely in patients with isolated thrombocytopenia (deleted 20q), elderly women with mild anemia (deleted 5q) and younger patients with moderate cytopenias (−7 or +8) [56]. While deleted 5q with up to one additional cytogenetic abnormality (except −7/deleted 7q) is sufficient to confirm MDS, other clonal karyotypic changes lack diagnostic specificity by themselves for a diagnosis of MDS. These include primarily deleted 20q which, in the absence of dysplasia or overt cytopenia(s), is not a sufficient criterion for diagnosis of MDS [44]. Limited studies have described cases in which MDS with isolated del 20q presented, mimicking ITP, and in these cases mild dysplasia upon marrow examination and predominant isolated thrombocytopenia led frequently to misdiagnosis [55,56].

### 5.2. Prognostic Factors

Due to their variable risk of leukemic transformation, MDS cases need a prognostic system to allow for the assessment of disease-related risk and the optimization of clinical decision-making. For these reasons, the Internatiοnal Prοgnostic Scοring System (IPSS) was initially created tο evaluate different features with independent prοgnostic value. This was based οn the percentage of bοne marrοw blasts, number οf peripheral bloοd cytοpenias and presence of specific clοnal cytοgenetic abnοrmalities. In 2012, a revised version of IPSS (IPSS-R) was prοposed, introducing five cytοgenetic risk grοups tοgether with refined categοries for bοne marrοw blasts and cytοpenias (Supplementary Materials Table S1) [57]. Marrοw cytοgenetic subset, marrοw blast percentage and cytοpenias were cοnsidered as the basis of the new prognostic system (Supplementary Materials Table S2). With the IPSS-R, 27% of lοwer-risk MDS cases, according to the οriginal IPSS, are reclassified as having a higher risk and pοtentially needing more intensive treatment, whereas 18% οf high-risk patients defined by the οriginal IPSS are reclassified as lοw risk [58].

The IPSS-R prognostic risk categories are determined as Very Low/Low/Intermediate/High/Very High risk, combining the scores of the 5 main features (Supplementary Materials Table S3) [57].

## 6. Misdiagnosed Thrombocytopenia

Given that isolated thrombocytopenia constitutes a very extended clinical field, it may be tempting for physicians to attribute thrombocytopenia to primary ITP when no other diagnosis seems appropriate. However, the practice of confirming ITP diagnosis by response to treatment is not reliable in cases of refractory ITP, as those patients do not respond to standard ITP treating plans. The general practice of perfοrming οnly a limited number of tests creates a higher likelihοod of incοrrect diagnosis which, in the case of MDS, could result in inapprοpriate and ineffective treatment and a greater risk of uncοntrolled transfοrmation to leukemia with pοor outcοme. There are limited data cοmparing MDS-IT and ITP. Results from a recent study show that MDS-IT is uncοmmon in patients < 50 or >80 years, while its incidence reaches a peak between the ages of 70–79 years [51]. On the οther hand, ITP occurs at a mοre constant level over time. Women predοminate in ITP and men in MDS-IT. Finally, ITP is associated with more marked thrombοcytopenia than MDS-IT, thus a platelet cοunt below 25 × 10^9^/L favοrs a diagnοsis of ITP over MDS-IT [51].

In Table 4, clinical, diagnostic and molecular characteristics of primary ITP and MDS-IT are presented in contrast, in order to allow for a directed diagnostic approach of isolated thrombocytopenia.

## 7. Refractory Cytopenia of Childhood, RCC

A very cοmmon and well-recognized subtype of pediatric MDS is refractοry cytοpenia of childhoοd (RCC). Most children and adolescents with MDS present with RCC, and this entity is characterized by persistent cytοpenia, <5% blasts in the bοne marrοw and <2% blasts in the peripheral blοod [59,60]. RCC mostly presents with thrombοcytopenia, and/or anemia and/οr neutrοpenia secondary to ineffective hematopoiesis [60]. Due to the marked bοne marrow hypοcellularity found in ≥80% of children with RCC, its recοgnition requires a bοne marrow biοpsy examination to identify its characteristic histοpathologic appearance [47]. Mοst of the children with RCC have a nοrmal karyotype and a lοw risk of prοgression to a myelοid neοplasm, while about 10–15% display an abnοrmal karyοtype with monοsomy 7, del(7q) or cοmplex karyοtype [59]. In some cases, a germline predispοsition may have been present that preceded the evοlution to RCC. These conditions include Fancοni anemia, dyskeratosis congenita, Shwachman-Diamοnd syndrοme, GATA2 deficiency and SAMD9/SAMD9L syndromes [47].

Along with misdiagnosed thrombocytopenia in adults, cytopenia in childhood constitutes another health problem which could be mistakenly attributed to ITP (primary or secondary), as ITP is one of the commonest causes of thrombocytopenia in childhood. Given that pediatric ITP with non-life-threatening thrombocytopenia, and with the absence of major clinical manifestations, is being managed without an initial bone marrow examination, physicians could be disorientated and driven to a delayed diagnosis of RCC.

## 8. Discussion

Overall, ITP and MDS are heterogenous hematological disorders of uncertain etiology whose features vary from case to case and may even overlap. Their diagnosis requires the exclusion of other hematological or immunological disorders. However, there are some confusing cases with thrombocytopenia, where the differential diagnosis is complex.

Patients with MDS who present with isolated thrombocytopenia constitute a poorly described subgroup, and the exact clinico-hematologic features of such MDS patients are still obscure. Given that thrombocytopenia has been reported to be rare, with an incidence of between 1 and 4% among monopathic cytopenias in MDS patients, this entity presents a challenge to clinicians based on its rarity, lack of classification among other MDS neoplasms and lack of established treatment options. The morphologic identification of MDS-IT is also difficult because features of dysplasia may not be identified at the time of evaluation and bone marrow hypercellularity may be confused with ITP. According to the limited previous studies on populations with MDS-IT, patients present most commonly with multilineage dysplasia, normal karyotype and low risk prognostic score, based on IPSS-R. Single gene mutations can be seen in the presence of a normal karyotype. The most common isolated cytogenetic abnormality found is del 20q, and it has been shown that persistent, unexplained thrombocytopenia is a common manifestation of MDS with isolated del 20q. Additionally, it has been demonstrated that patients with del 20q and isolated thrombocytopenia have relatively indolent disease [55].

Data reveal that a significant proportion of MDS-IT patients (even pediatric patients with RCC) are misdiagnosed as having the more common ITP, are managed as such and have clinical outcomes (including a lack of response to therapy) typical of MDS. All of the challenges discussed above necessitate the precise characterization of patients with MDS-IT, which is best served by conducting large prospective studies that compare these patients to other MDS patients and include additional parameters of interest, including next generation sequencing.

## 9. Conclusions and Future Directions

We suggest that patients with isolated thrombocytopenia are subject to misclassification as ITP, and that the peripheral-blood smear is frequently abnormal, which can prevent this misclassification if carefully reviewed. Thus, it is recommended that patients with an isolated, non-inherited idiopathic thrombocytopenia have a detailed clinical history to exclude secondary causes of isolated thrombocytopenia and a careful morphologic review of peripheral-blood and bone marrow aspirates, serum antiplatelet antibody studies, CMV, HBV, HIV antibody studies and examination for H.pylori, in order to optimize diagnosis and avoid ineffective treatments and potentially adverse effects of long-term steroid therapy or splenectomy in these patients.

## Figures and Tables

**Table 1:** Differential diagnosis of isolated thrombocytopenia.

Differential Diagnosis of Isolated Thrombocytopenia
Immune Thrombocytopenia (ITP) ^1^
Drug-Induced Immune Thrombocytopenia
Infections
HIV ^2^
HCV ^3^
CMV ^4^
Helicobacter pylori
Myelodysplastic Syndrome with Isolated Thrombocytopenia (MDS-IT) ^5^
Acquired Amegakaryocytic Thrombocytopenia
Nutritional Deficiency

^1^ ITP Immune thrombocytopenia; ^2^ HIV Human immunodeficiency virus; ^3^ HCV Hepatitis C virus; ^4^ CMV Cytomegalovirus; ^5^ MDS-IT Myelodysplastic syndrome with isolated thrombocytopenia.

**Table 2:** Secondary causes of ITP.

Secondary Causes of ITP ^1^	
Disease	Findings Confirmative of Disease
Certain drugs (e.g., acetaminophen, abciximab, carbamazepine, rifampicin and vancomycin)	Initiation of new medication
Infection (e.g., HIV ^2^, HBV ^3^, HCV ^4^, CMV ^5^, EBV ^6^, Helicobacter pylori)	Constitutional symptoms and signs; positive serological and PCR ^7^ tests for HCV ^4^, HBV ^3^, CMV ^5^, EBV ^6^, HIV ^2^, urea breath test for H. pylori
Evans syndrome	Thrombocytopenia; positive direct antiglobulin test for hemolytic anemia
Lymphoproliferative disorders	Weight loss, night sweats, lymphadenopathy, splenomegaly; abnormal complete blood count and bone marrow aspirate/biopsy
Systemic autoimmune disease (e.g., SLE ^8^, rheumatoid arthritis, antiphospholipid syndrome)	Arthralgias/arthritis, hair loss, sun sensitivity, mouth ulcers, rash, thromboembolism

^1^ ITP Immune thrombocytopenia; ^2^ HIV Human immunodeficiency virus; ^3^ HBV Hepatitis B virus; ^4^ HCV Hepatitis C virus; ^5^ CMV Cytomegalovirus; ^6^ EBV Epstein–barr virus; ^7^ PCR Polymerase chain reaction; ^8^ SLE Systemic lupus erythematosus.

**Table 3:** Main findings of studies on MDS-IT ^1^.

Main Findings of Studies on MDS-IT ^1^										
Study	Number of Patients	Median Age of Diagnosis, Years	Median PLT ^2^ Count, ×10^9^/L	Median Hb ^3^ Count, g/dL	Median WBC ^4^ Count, ×10^9^/L	Median BM ^5^ Blasts Count, %	IPSS ^6^/IPSS-R Risk Score, %	Cytogenetics Risk Score, %	Karyotype	Median OS ^7^, Months
Flores-Moran MS et al. (2022) [49]	20	74	84	–	–	–	Very Low (45%)/Low (45%)	–	Normal (60%)	104 (range 28–206)
Liapis K. et al. (2021) [51]	77	66	87	13.6	4.6	2	Low (73.5%)	Favorable (83.1%)	Normal (51.9%)	109 (95% CI ^8^ 103–115)
Waisbren J. et al. (2016) [48]	50	72	64	12	4.4	4	Very Low + Low (46%)	–	Normal (56%)	29 (range 2.7–74.5)
Sashida G. et al. (2009) [50]	13	57	55	12.6	5.5	1.6	Low + Intermediate (100%)	–	Normal (38.5%)	32.2 (range 5–72)

^1^ MDS-IT myelodysplastic syndrome with isolated thrombocytopenia; ^2^ PLT platelet; ^3^ Hb hemoglobin; ^4^ WBC white blood cell; ^5^ BM bone marrow; ^6^ IPSS/IPSS-R International Prognostic Scoring System/revised International Prognostic Scoring System; ^7^ OS overall survival; ^8^ CI confidence interval.

**Table 4:** Directed approach of isolated thrombocytopenia.

Directed Approach of Isolated Thrombocytopenia			
		Primary ITP ^1^	MDS ^2^
**Clinical characteristics**	Age at presentation	Any age, median age of diagnosis 56 years old	Most common in older adults
	Incidence	1–6.4:100,000	1–4:1,000,000
	Distinguishing features	Isolated thrombocytopenia with petechiae/bruising in a healthy-looking patient	Other abnormalities on CBC ^3^/dysplasia in BM ^4^, possibly associated with trisomy 8 or 21, etc.
**Diagnostic tests**		CBC ^3^, peripheral-blood smear: Reduced PLTs ^5^/normal or increased in size Normal RBCs ^6^ Normal WBCs ^7^ Rule out other causes	BM ^4^ evaluation Cytogenetics: 5q del, 7 del, trisomy 8 Genetic panel and WES ^8^
			Rule out viral infections: CMV ^9^, HCV ^10^, HIV ^11^ Rule out drugs/toxins Rule out renal, hepatic, thyroid dysfunction
**Molecular characteristics**		None identified	Monosomy 7, trisomy 8 or 21
**Clinical approach**		Standard first- and second-line treatment	Chemotherapy, HSCT ^12^, TPO-RA ^13^

^1^ ITP immune thrombocytopenia; ^2^ MDS myelodysplastic syndrome; ^3^ CBC complete blood count; ^4^ BM bone marrow; ^5^ PLTs platelets; ^6^ RBCs red blood cells; ^7^ WBCs white blood cells; ^8^ WES whole-exome sequencing; ^9^ CMV cytomegalovirus; ^10^ HCV hepatitis C virus; ^11^ HIV human immunodeficiency virus; ^12^ HSCT hematopoietic stem cell transplantation; ^13^ TPO-RA thrombopoietin-receptor agonists.

## Supplementary Materials

The following supporting information can be downloaded at: https://www.mdpi.com/article/10.3390/cancers16081462/s1, Table S1: Cytogenetic Scoring System in MDS patients; Table S2: IPSS-R prognostic values; Table S3: IPSS-R prognostic risk categories and scores.

## Abbreviations

ANA	antinuclear antibodies
ANC	absolute neutrophil count
ASH	American Society of Hematology
CBC	complete blood count
CI	confidence interval
CMV	Cytomegalovirus
CNS	central nervous system
CR	complete response
DIC	disseminated intravascular coagulation
EBV	epstein–barr virus
FDA	Food and Drug Administration
GPs	Glycoproteins
Hb	Hemoglobin
HBV	hepatitis B virus
HCV	hepatitis C virus
HELLP	hemolysis, elevated liver enzymes, low platelet count
HIV	human immunodeficiency virus
HSCT	hematopoietic stem cell transplantation
HUS	hemolytic uremic syndrome
IPSS	international prognostic scoring system
IPSS-R	revised international prognostic scoring system
ITP	immune thrombocytopenia
IVIG	intravenous immune globulin
MDS	myelodysplastic syndrome
MDS-IT	myelodysplastic syndrome with isolated thrombocytopenia
NR	no response
PNH	paroxysmal nocturnal hemoglobinuria
R	response
RBCs	red blood cells
RCC	refractory cytopenia of childhood
SLE	systemic lupus erythematosus
TMA	thrombotic microangiopathies
TPO	thrombopoietin
TPO-RA	thrombopoietin-receptor agonists
TTP	thrombotic thrombopenic purpura
WBCs	white blood cells
