# Supplementary material for: The Challenge for a Correct Diagnosis of Refractory Thrombocytopenia: ITP or MDS with Isolated Thrombocytopenia?
Source: Cancers (Basel). 2024 Apr 11;16(8):1462. doi: 10.3390/cancers16081462 (PMC11048195; doi:10.3390/cancers16081462)
Supplement: Supplementary file 1 [file cancers-16-01462-s001.zip › cancers-2918514-supplementary.pdf]

Supplementary Materials:

Table S1. Cytogenetic Scoring System in MDS patients

**Cytogenetic Scoring System in MDS[1] Patients**				
**Prognostic cytogenetic subgroups**	**Percentage of patients, %**	**Karyotype**	**Median survival, years**	**Median time to 25% AML[2] evolution, years**
Very Good	4	−Y, del(11q)	5.4	NR[3]
Good	72	Normal, del(5q), del(12p), del(20q), double including del(5q)	4.8	9.4
Intermediate	13	del(7q), +8, +19, i(17q), any other single or double independent clones	2.7	2.5
Poor	4	−7, inv(3)/t(3q)/del(3q), double including −7/del(7q), complex: 3 abnormalities	1.5	1.7
Very Poor	7	Complex: > 3 abnormalities	0.7	0.7

[1]MDS myelodysplastic syndrome; [2]AML acute myeloid leukemia; [3]NR not reached

Table S2. IPSS-R prognostic values

IPSS-R[1] prognostic score values							
**Prognostic value**	**0 points**	**0.5 points**	**1 points**	**1.5 points**	**2 points**	**3 points**	**4 points**
Cytogenetics*	Very Good	–	Good	–	Intermediate	Poor	Very Poor
BM[2] blasts, %	≤ 2	–	> 2 to < 5	–	5 to 10	> 10	–
Hemoglobin, g/dL	≥ 10	–	8 to < 10	< 8	–	–	–
Platelets, $\times 10^9$/L	≥ 100	50 to < 100	< 50	–	–	–	–
ANC[3], $\times 10^9$/L	≥ 0.8	< 0.8	–	–	–	–	–

*According to Table 4
– indicates not applicable
[1]IPSS-R revised International Prognostic Scoring System; [2]BM bone marrow; [3]ANC absolute neutrophil count

TableS 3. IPSS-R prognostic risk categories and scores

IPSS-R[1] prognostic risk categories/scores	
**Risk category**	**Risk score**
Very Low	≤ 1.5
Low	> 1.5-3
Intermediate	> 3-4.5
High	> 4.5-6
Very High	> 6

[1]IPSS-R revised International Prognostic System
